# Quinolone and macrolide resistance in Campylobacter jejuni and C. coli: resistance mechanisms and trends in human isolates.

**DOI:** 10.3201/eid0701.010104

**Published:** 2001

**Authors:** J Engberg, F M Aarestrup, D E Taylor, P Gerner-Smidt, I Nachamkin

**Affiliations:** Department of Gastrointestinal Infections, Division of Diagnostics, Statens Serum Institut, Artillerivej 5, DK-2300 Copenhagen S, Denmark. eng@ssi.dk

## Abstract

The incidence of human Campylobacter jejuni and C. coli infections has increased markedly in many parts of the world in the last decade as has the number of quinolone-resistant and, to a lesser extent, macrolide-resistant Campylobacter strains causing infections. We review macrolide and quinolone resistance in Campylobacter and track resistance trends in human clinical isolates in relation to use of these agents in food animals. Susceptibility data suggest that erythromycin and other macrolides should remain the drugs of choice in most regions, with systematic surveillance and control measures maintained, but fluoroquinolones may now be of limited use in the empiric treatment of Campylobacter infections in many regions.


### Quinolone and Macrolide Resistance in *Campylobacter jejuni* and *C. coli*: Resistance Mechanisms and Trends in Human Isolates

**Jørgen Engberg,* Frank M. Aarestrup,† Diane E. Taylor,‡ Peter Gerner-Smidt,* and Irving Nachamkin§**
*Statens Serum Institut, Copenhagen, Denmark; †Danish Veterinary Laboratory, Copenhagen, Denmark; ‡ University of Alberta, Edmonton, Alberta, Canada; §University of Pennsylvania, Philadelphia, Pennsylvania, USA

The incidence of human *Campylobacter jejuni* and *C. coli* infections has increased markedly in many parts of the world in the last decade as has the number of quinolone-resistant and, to a lesser extent, macrolide-resistant *Campylobacter* strains causing infections. We review macrolide and quinolone resistance in *Campylobacter* and track resistance trends in human clinical isolates in relation to use of these agents in food animals. Susceptibility data suggest that erythromycin and other macrolides should remain the drugs of choice in most regions, with systematic surveillance and control measures maintained, but fluoroquinolones may now be of limited use in the empiric treatment of *Campylobacter* infections in many regions.

*Campylobacter jejuni* subsp. *jejuni* (*C. jejuni*) and *C. coli* have been recognized since the late 1970s as important agents of gastrointestinal infections throughout the world; in the United States, these infections affect approximately 1% of the population each year (1). Contaminated food is the usual source of human infections; therefore, the presence of fluoroquinolone- and macrolide-resistant strains in the food chain has raised concerns that the treatment of human infections will be compromised. Most cases of *Campylobacter* enteritis do not require antimicrobial treatment, being brief, clinically mild, and self-limiting (2-4). However, a substantial proportion of these infections require treatment. These include severe and prolonged cases of enteritis, septicemia, and other extraintestinal infections. Erythromycin has been the most commonly used agent for treating *Campylobacter* enteritis (2,5).

In the 1980s, the introduction of fluoroquinolones, which are effective against most major pathogens causing bacterial enteritis, offered a new approach to antibiotic intervention (6). Fluoroquinolones initially had good in vitro activity for thermophilic *Campylobacter* species, as well as for members of the family of Enterobacteriaceae.

Early clinical trials of both community-acquired acute diarrhea and traveler's diarrhea caused by *Campylobacter* demonstrated that patients treated with a fluoroquinolone had good clinical response (6,7). It soon became apparent, however, that resistance in *Campylobacter* spp. could arise in vivo, sometimes after only one or two administrations of fluoroquinolones (8). Moreover, Endtz and colleagues (9) reported as early as 1991 that the emergence of quinolone-resistant *C. jejuni* and *C. coli* isolated from humans in the Netherlands coincided with the introduction of fluoroquinolones in veterinary medicine.

Fluoroquinolone resistance in *Campylobacter* from food animals is now recognized as an emerging public health problem. Smith et al. from Minnesota (10) found that patients infected with resistant *C. jejuni* had longer duration of

Address for correspondence: Jørgen Engberg, Department of Gastrointestinal Infections, Division of Diagnostics, Statens Serum Institut, Artillerivej 5, DK-2300 Copenhagen S, Denmark; fax: 45-3268-8238; e-mail: eng@ssi.dk.

diarrhea than patients with fluoroquinolone-sensitive isolates. As *Campylobacter* infections can be serious in immunocompromised patients, the identified treatment failure raises the concern that fluoroquinolone-resistant strains may increase *Campylobacter*-associated deaths in this group of patients.

### Mechanism of Macrolide Resistance in *Campylobacter*

Erythromycin binds to the ribosome but, unlike larger macrolides, appears to cause dissociation of the peptidyl-tRNA, rather than blocking the peptidyltransferase activity (11).

In *C. jejuni* and *C. coli*, erythromycin resistance is chromosomally mediated and is due to alteration of the ribosome (12); the resistance mechanism is not consistent with presence of an rRNA methylase, modification of the antibiotic, or efflux (13). Whole ribosomes or 50S subunits were purified from erythromycin-resistant strains and shown to bind much less erythromycin than ribosomes from sensitive strains. In a closely related bacterium, *Helicobacter pylori*, resistance to clarithromycin is due to an alteration of one of two adenine residues in the 23S rRNA at the erythromycin-binding site (14). Sequencing of the 23S rRNA genes from erythromycin-resistant *Campylobacter* spp. identified mutations at these same sites, which are most probably responsible for resistance (Figure 1) (15).

| **Resistance** | | **Mutation** |
|---|---|---|
| **Macrolide** | ⟶ | **Domain V of 23S rRNA**<br>**A2058 → G**<br>**A2059 → G** |
| **Fluoroquinolone** | ⟶ | ***gyr*a → Thr - 86**<br>**(higher MIC)**<br>**Thr - 90**<br>**Ala - 70**<br>**(lower MIC)** |
| | ⟶ | ***gyr*a → Thr - 86**<br>**+**<br>***par*c→ Arg - 139**<br>**(highest MIC)** |

Figure 1. Macrolide and fluoroquinolone resistance mechanisms reported in *Campylobacter* species. For macrolide resistance, mutations are at either position shown (*Escherichia coli* coordinates) in up to all three copies of ribosomal RNA (14,15, and CA Trieber & DE Taylor, unpub. data). Fluoroquinolone resistance depends on a mutation in the quinolone resistance determining region of DNA gyrase A (*Gyr*A). For typical MICs see text and references 16-18. The strains with highest resistance levels had mutations in both *Gyr*A and topoisomerase IV *Par*C.

### Mechanism of Fluoroquinolone Resistance in *Campylobacter*

Fluoroquinolone resistance in *C. jejuni* appears to be due most often to mutations in the genes encoding subunits of DNA gyrase (*gyr*A) and only occasionally to topoisomerase IV (*par*C) (Figure 1). DNA gyrase purified from quinolone-resistant mutants of *C. jejuni* was 100-fold less sensitive to inhibition by quinolones than the wildtype gyrase (19). Cloning and sequencing of the *C. jejuni gyr*A gene demonstrate that mutations in *gyr*A at positions Thr-86, Asp-90, and Ala-70 were responsible for resistance (16,17). Mutations at Thr-86 are associated with higher level resistance to nalidixic acid (MIC 64-128 µg/mL) and ciprofloxacin (MIC 16-64 µg/mL) than mutations at Asp-90 or Ala-70. *C. jejuni* isolates resistant to even higher levels of quinolones (ciprofloxacin MIC of 125 µg/mL) carry two mutations, one in *gyr*A Thr-86 and the other in the topoisomerase IV subunit *par*C at Arg-139 (18).

Evidence of efflux of fluoroquinolones in *C. jejuni* (20) also exists. Passage of the bacteria on pefloxacin-containing agar has led to the isolation of a fluoroquinolone-resistant strain. This strain was also resistant to tetracycline, erythromycin, chloramphenicol, and several β-lactams. The pefloxacin-resistant strain carried a mutation at Thr-86 of *gyr*A, likely responsible, in part, for fluoroquinolone resistance. Broad-specificity efflux pumps in *C. jejuni,* which cause fluoroquinolone resistance, have not yet been shown to be clinically relevant.

### Use of Macrolides and Quinolones in Food Animals

Antibiotics of the macrolide-lincosamide group have been used in treating food animals worldwide for several decades. The most commonly used agents have been lincomycin and tylosin for controlling dysentery and *Mycoplasma* infections in swine and spiramycin for treating mastitis in cattle. For the past 20 years, tylosin has also been the most commonly used agent for growth promotion in swine production worldwide, whereas spiramycin has been commonly used in poultry. The use of macrolides for growth promotion has been banned in all European Union countries since July 1999.

Several fluoroquinolones are available for treating food animals, such as poultry, cattle, pigs, and fish, in many countries. While information on global use is limited, worldwide use in food animals was estimated at 120 tons in 1997; use in humans has been estimated at more than 800 tons (21). Data are available only for the year of veterinary licensing of fluoroquinolones by country (Table 1). Licensing for use does not necessarily mean that the drug is actually used, so even these data have to be considered with caution. However, quinolone treatment of *Campylobacter*-colonized broiler chickens has induced quinolone resistance under experimental conditions (23).

### Macrolide and Quinolone Resistance in Isolates from Food Animals and Foods of Animal Origin

*Campylobacter* is carried in the intestinal tract of wild and domestic animals and, as result

Table 1. Veterinary licensing of fluoroquinolones in selected countries

| Country | Substance | Licensing year | Animal species |
|---|---|---|---|
| Austria (22) | Enrofloxacin | 1992 | Cattle, pigs, poultry |
| | Danofloxacin | 1996 | Poultry |
| | Difloxacin | 1998 | Poultry |
| Canada[a] | Enrofloxacin | 1987 (withdrawn in 1997) | Turkey (egg dip) |
| Denmark (22) | Enrofloxacin | 1991 | Cattle, pigs, poultry |
| | Danofloxacin | 1993 | Poultry |
| | Difloxacin | 1998 | Poultry, turkey |
| | Marbofloxacin | 1998 | Cattle, pigs, dogs, cats |
| Finland (22) | Enrofloxacin | 1992 (oral use withdrawn in 1999) | Pigs |
| | Difloxacin | 1998 | Poultry |
| France (22) | Enrofloxacin | 1991 | Cattle, poultry |
| | Danofloxacin | 1996 | Cattle |
| | Marbofloxacin | 1993 | Cattle |
| | Difloxacin | 1998 | Poultry |
| Italy (22) | Enrofloxacin | 1989 | Cattle, pigs, poultry |
| | Difloxacin | 1998 | Poultry |
| Japan[b] | Enrofloxacin | 1991 | Cattle, poultry |
| | | 1992 | Pigs |
| | Danofloxacin | 1992 | Poultry |
| | | 1993 | Cattle, pigs |
| | Ofloxacin | 1992 | Poultry |
| | Orbifloxacin | 1993 | Cattle, pigs |
| | Difloxacin | 1996 | Pigs |
| | Norfloxacin | 1998 | Poultry |
| Netherlands (22) | Enrofloxacin | 1987 | Cattle, pigs, poultry |
| | Difloxacin | 1998 | Poultry |
| Spain (22) | Enrofloxacin | 1986 | Cattle, pigs, poultry |
| | Difloxacin | 1998 | Poultry |
| United Kingdom (22) | Enrofloxacin | 1993 | Cattle, pigs, poultry |
| | Danofloxacin | 1993 | Poultry |
| | Marbofloxacin | 1995 | Cattle |
| | Difloxacin | 1998 | Poultry |
| USA[c] | Enrofloxacin | Approx. 1987-88 | Dogs, cats |
| | | 1996 | Poultry |
| | Sarafloxacin | 1999 | Cattle |
| | | 1995 | Poultry |

[a]RJ Irwin, Health Canada, 1999. pers. comm.
[b]Y Tamura, National Veterinary Assay Laboratory, Japan, 1999. pers. comm.
[c]JL Watts. Pharmacia/Upjohn, Kalamazoo, Michigan, 1999. pers. comm.

of fecal contact during processing, frequently contaminates foods derived from animals. *C. jejuni* is predominant in broilers and cattle but is infrequent in pigs (where *C. coli* predominates) (24). In food animals, the prevalence of resistance to erythromycin is generally higher in *C. coli,* in particular in *C. coli* isolates from pigs, than in *C. jejuni* (24-26). In a recent study from Spain (27), rates of erythromycin and quinolone resistance in *C. coli* from pigs were 81% and 100%, respectively. High erythromycin resistance in pigs may be related to extensive veterinary use of macrolides (5,28).

In food products of animal origin, the occurrence of *Campylobacter* is much higher in poultry than in other categories, e.g., pork or beef (29). Therefore, *Campylobacter* resistance data are primarily based on poultry products, especially broiler meat. For a number of countries, fluoroquinolone-resistance rates are similar in isolates from poultry products and humans (10,25,27,30-32). In the United Kingdom, enrofloxacin (a derivative of ciprofloxacin) was first licensed in late 1993; before then, domestically bred chickens were less frequently infected with quinolone-resistant campylobacters than imported chicken products. Using a simple model, researchers were able to correlate the previously observed resistance percentage in domestically acquired cases with estimates of the amount of imported chicken consumed in the United Kingdom (32). In recent data from Spain and Taiwan, rates of erythromycin resistance were 17% and 17%, respectively, in *C. jejuni* isolated from foods, whereas for *C. coli* the figures were 50% and 83%, respectively (27,31).

### Transmission of Resistant *Campylobacter* from Animals to Humans

Campylobacteriosis is primarily a zoonosis. Evidence to indicate that fresh raw meat, especially poultry, is a major source of infection is ample, even though other sources such as raw milk, water, and pets may contribute to human infection (1,5,33-38).

Studying the transmission of antimicrobial resistance from animals (especially poultry to humans) has been difficult because the chain of transmission is often complex. The number of macrolide- and fluoroquinolone-resistant isolates from humans is influenced by several factors including veterinary use of macrolides (approved for use as antimicrobial growth promoters or as therapeutic drugs) and fluoroquinolones (only approved as therapeutic drugs) at a given location (24,39); association with recent or current antimicrobial treatment of patients; the origin of isolates (children vs. adults; inpatients vs. outpatients); travel (10,40-45); and sampling strategy and susceptibility testing procedures (no consensus as to method, media, culture conditions, or breakpoints [43,46]). These factors stress the need for cautious interpretation and comparison of data from different centers. However, several studies have shown that food animals can be a substantial source of infection in humans and that the same serotypes and genotypes can be isolated from humans and food animals (29,36,37,47-49). DNA profiling of Danish *C. jejuni* serotype O:2 strains using pulsed-field gel electrophoresis with four restriction enzymes identified common genotypes in humans, poultry, cattle and swine (SLW On, EM Nielsen, and J Engberg, unpub. data). Typing data on resistant isolates is sparse, but Smith and colleagues (10) found DNA fingerprints of quinolone-resistant *C. jejuni* from U.S.-produced poultry identical to those of resistant *C. jejuni* from domestically acquired infections in humans. Therefore, the susceptibility of humans strains originating in animals to antibiotics can be related to the exposure of animal strains to antibiotic agents used in farming.

### Is There a Link Between Macrolide and Fluoroquinolone Use in Humans and Resistant *Campylobacter* Infections?

Some investigators suggest that resistance in *C. jejuni* and *C. coli* is driven by use of antibiotics for treating human infections rather than by veterinary use. Induction of macrolide resistance during treatment has been reported infrequently (50). However, induction of macrolide resistance may play a role in areas with a large reservoir of asymptomatic *Campylobacter* carriers and frequent use of macrolides in humans.

Induction of fluoroquinolone resistance during treatment is well recognized and often reported (8,51-53). A predicted 10% of patients treated with a fluoroquinolone for *Campylobacter* enteritis harbor quinolone-resistant *Campylobacter* strains (6). Recently, Ellis-Pegler (53) found that fluoroquinolone resistance developed in 18% to 28% of patients in their prospective trial. Development of resistance has been reported within 24 hrs of treatment, but prolonged therapy, e.g., in immunosuppressed patients, is also a risk factor (52,54).

Smith et al. (10) showed that use of a quinolone before culture accounted for a maximum of 15% of resistant isolates during 1996 to 1998. In addition, an increasing number of reports claim that fluoroquinolone-resistant strains have been isolated from patients who had not received medical treatment, suggesting that strains were already fluoroquinolone resistant before causing the infection (7,31,32,55-57). Since human-to-human transmission of *C. jejuni* and *C. coli* is rare (9), patients infected with resistant *Campylobacter* are not an important source of resistant *Campylobacter* for other humans.

Before fluoroquinolones were introduced in veterinary medicine, they were widely used in human medicine in a number of countries, including the Netherlands and the United States (since 1985 and 1987, respectively), without emergence of quinolone resistance in *Campylobacter* in humans. In contrast, emerging quinolone resistance in humans often coincides with or follows the approval of fluoroquinolones in animal husbandry (Table 1, Figure 2). Thus, while human macrolide and fluoroquinolone use contributes to the increase in resistance in humans, their relative contribution to increase in resistance compared to the use of these agents in husbandry appears to be small.

### Frequency of Macrolide Resistance in Human Isolates

Data on erythromycin and azithromycin resistance in *C. jejuni*, *C. coli,* and the two organisms combined, isolated from humans around the world since 1989, differ by country

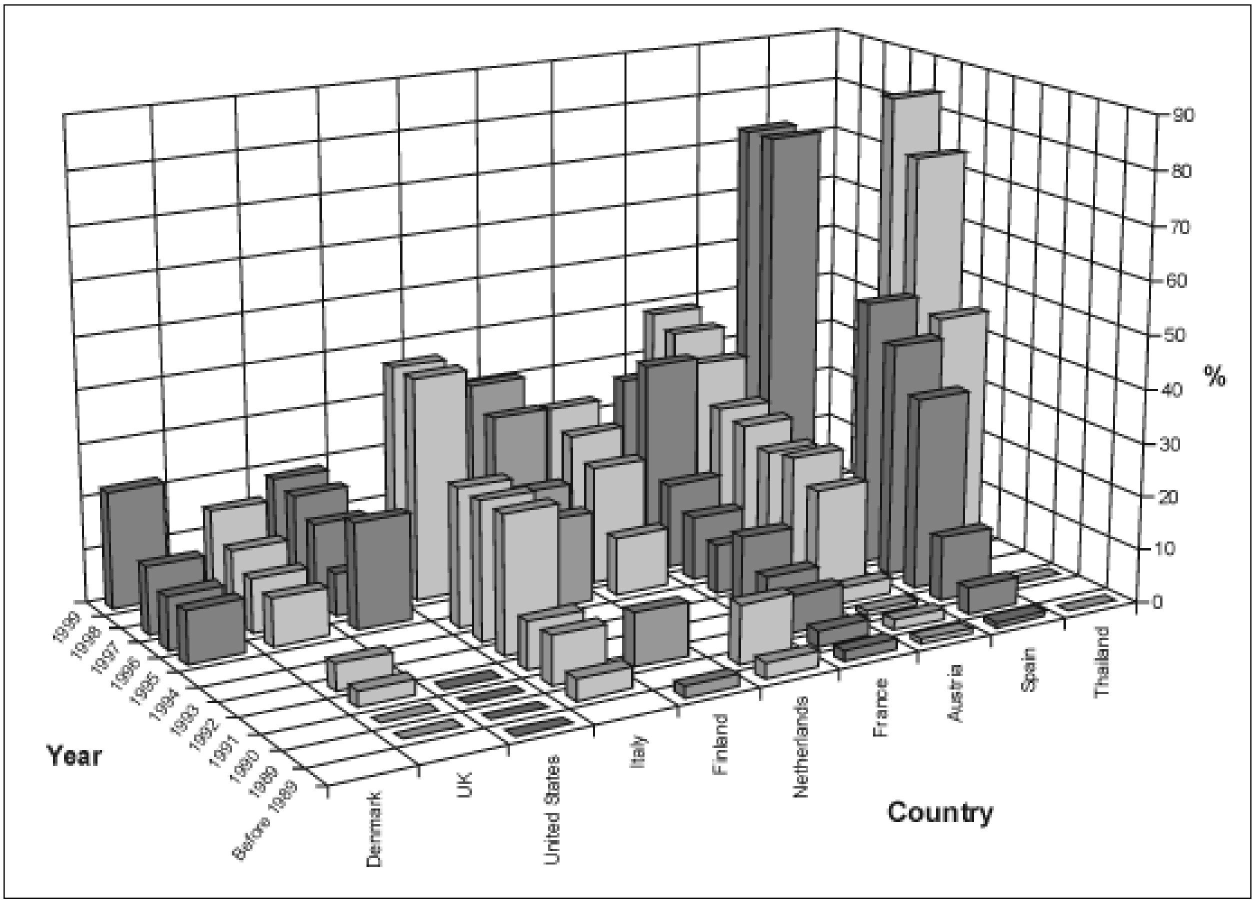

Figure 2. Trends for quinolone resistance rates (in percentages) among *Campylobacter coli* and *C. jejuni* combined from human sources around the world. The bars represent both nalidixic acid and fluoroquinolone resistance and are based on mean values of resistance from numerous reports (9,17,24,27,39,43,56-58,61-64,72-75,78,88, plus pers. comm. from Feirel G and Rautelin H, and unpub. data from Nachamkin I).

and species (Table 2). Almost all studies report a higher frequency of erythromycin resistance in *C. coli* than in *C. jejuni* (0% to 11% in *C. jejuni* vs. 0% to 68.4% in *C. coli)*. Trends over time for erythromycin resistance show stable and low rates in Japan, Canada, and Finland, but recent development of resistance in Thailand and Sweden (45,73).

### Trends over Time for Quinolone Resistance

Resistance to fluoroquinolones in *Campylobacter* has clearly increased over the past decade in many parts of the world (Figure 2). Before 1989, resistance was rare. With the introduction of enrofloxacin in veterinary medicine (Table 1) and (probably less important) fluoroquinolones in human medicine in mainland Europe (the Netherlands, Finland, France, and Spain), a rapid emergence of quinolone resistance in *Campylobacter* isolates from patients was noted (8,9,43,55,64,89,90).

Surveillance data on resistance rates in human isolates from Asia soon indicated an equal increase (84,91). Quinolones were approved for veterinary use in the United Kingdom and the United States in late 1993 and 1995, respectively; reports from these areas now show increasing quinolone-resistance profiles (10,39,88).

In the latest data from Taiwan, Thailand, and Spain, rates of fluoroquinolone resistance in *C. jejuni*, or *C. jejuni* and C. *coli* combined were 56.9%, 84%, and 75% to 88%, respectively (27,31,40,73). In these regions, where quinolone resistance is highly endemic and *Campylobacter* spp. predominate, fluoroquinolones cannot be recommended for community-acquired bacterial diarrhea. Although lower frequencies are reported from other regions, recent trends show a clear tendency of emerging quinolone resistance in many countries. Quinolone resistance in human isolates often coincides with or follows the approval of fluoroquinolones for use in animal husbandry (Table 1, Figure 2), although some differences in resistance rates between countries may be explained by differences in association with foreign travel, commerce, methods of testing, and surveillance activity.

### Multidrug Resistance

Multidrug resistance to macrolides and fluoroquinolones must be considered highly undesirable in *Campylobacter* as these two classes are generally advocated as first- and second-line drugs for antimicrobial treatment of *Campylobacter* enteritis.

Additional resistance to other relevant therapeutic agents poses a risk when there is no effective antimicrobial regimen for *Campylobacter* infections. Recently, Hoge et al. (73) found 100% co-resistance between Thai isolates resistant to azithromycin and ciprofloxacin in the last 2 years

Table 2. Erythromycin and azithromycin resistance rates (%) in *Campylobacter* in humans, worldwide, since 1989

| Country | *C. jejuni* | *C. coli* | *C. jejuni* and *C. coli* | Reference |
|---|---|---|---|---|
| Austria | 0.7 | 5.5 | <1-1.4 | (58 & pers. comm.[a]) |
| Canada | 0-3.3 | - | - | (59,60) |
| Denmark | 0 | 14.0 | 0-4 | (24,61,62) |
| Finland | - | - | <1-3 | (43,63, & pers. comm.[b]) |
| France | 1.1 | 12.2 | 3.5 | (64) |
| Hungary | - | - | 0 | (65) |
| Italy | 1.2-6 | 16-68.4 | 7.8-11.6 | (66-69) |
| Japan | 0.8 | - | - | (66) |
| New Zealand | - | - | 1.5 | (70) |
| Singapore | - | - | 51 | (71) |
| Spain | 0-11.0 | 0-35.0 | 3.2-7.3 | (17,27,56,57,72) |
| Sweden | 6.4[c] | 11.1[c] | 7.3[c] | (44) |
| Taiwan | 10.0 | 50.0 | 18.3 | (31) |
| Thailand | - | - | 0-31.0 | (73,74) |
| United Kingdom | 1 | 13 | 1.8 | (75) |
| United States | 0-7.8 | - | - | (10,76-78, & unpub. data[d]) |

[a]G Feierl, 2000, pers. comm.
[b]H Rautelin, 1999, pers. comm.
[c]90% of isolates were acquired abroad.
[d]I Nachamkin, 2000, unpub. data.

of surveillance. In addition, the level of tetracycline and ampicillin resistance in Thailand is so high that these agents now have no role in the treatment of *Campylobacter* or noncholera diarrhea. Li et al. (31) reported that concomitant resistance rates among nalidixic acid-resistant *C. jejuni* isolates from their patients (exclusively children) were as follows: gentamicin 2%, erythromycin 12%, clindamycin 12%, tetracyline 97%, and ciprofloxacin 66%. All of these human erythromycin-resistant *C. jejuni* isolates and 90% of the *C. coli* isolates were concomitantly resistant to clindamycin.

### Consequences of Resistance for the Clinical Decision-making Process

Distinguishing infections caused by different enteric pathogens is seldom possible, so antimicrobial-drug use in the clinical setting is not confined to the treatment of *Campylobacter* spp. but rather to empiric treatment of community-acquired diarrhea in general. Increased rates of resistance have also been reported from nontyphoidal salmonellae (25,92), and documented failures in the treatment of human *Salmonella* infections have been described (93). Therefore, having continuous information on the resistance patterns of the most common bacteria causing gastrointestinal infections is critical.

### Control Measures

Surveillance of resistance in *Campylobacter* is of paramount importance when fluoroquinolones are used to treat human infections. Systematic surveillance and timely reporting of antibiotic resistance patterns in *C. jejuni* and *C. coli* and other enteric pathogens from different regions should become a high priority. The principal purpose of monitoring antimicrobial resistance trends in enteric pathogens is to provide clinicians with data that can be used to select appropriate treatment regimens. Surveillance should emphasize antibiotics that are being used routinely to treat diarrhea, as well as any alternatives, such as fluoroquinolones, macrolides, and gentamicin. Equally important is the accessibility of the data to those providing primary care. For quinolones, quantitative nalidixic acid susceptibility data are more sensitive than fluoroquinolone susceptibility data for detecting common first-step mutations causing reduced susceptibility.

To circumvent the development of resistance, we have two options: infection control (zoonoses control) and prudent use of antibiotics. Improved infection control strategies along the chain "stable to the table" and guidelines for prudent use of antimicrobial agents in food animal production should be developed (94,95). To prevent further development of resistance in *Campylobacter,* limiting the use of macrolides and fluoroquinolones for food animals as much as possible is recommended. In Denmark, fluoroquinolones are not essential for treatment of any type of infection in food animals, according to surveillance performed by the Danish Veterinary Laboratory, and their use is only recommended on the rare occasion when no other therapeutic option is available (22). Because of the selection for resistance, the use of macrolides for growth promotion has been banned in all European Union countries since July 1999. The effect on the occurrence of resistance in bacteria in food animals is still not known. However, preliminary results suggest that macrolide resistance in *C. coli* from pigs in Denmark has decreased along with the decreased use of tylosin (FM Aarestrup, unpub. data).

### Conclusions

Review of in vitro macrolide and quinolone resistance prevalence and trends in *Campylobacter* isolated from humans showed a temporal relationship between use of quinolones in food animals and resistant *Campylobacter* isolates in humans. The public health effects of antibiotic use in agricultural practice, including resistance of *C. jejuni* and *C. coli* to macrolides and quinolones, should be estimated. Adequate actions for control are strongly needed in both veterinary and human medicine. The public health issue of resistance in *Campylobacter* has global dimensions because of ever-increasing international trade and travel.

This work was supported in part by the Natural Sciences and Engineering Research Council of Canada to D.E.T., a medical scientist with the Alberta Heritage Foundation for Medical Research.

Dr. Engberg is a physician at the Danish national reference laboratory for enteric pathogens at Statens Serum Institut. His research interests focus on the epidemiologic, antimicrobial susceptibility, and molecular typing aspects of *Campylobacter*.

### References

1. Tauxe RV. In: Nachamkin I, Blaser MJ, Tompkins LS, editors. *Campylobacter jejuni*: current status and future trends. Washington: American Society for Microbiology; 1992. p. 9-19.
2. Blaser MJ, Mandell GL, Bennett JE, Dolin R, editors. Principles and practice of infectious diseases. 4th ed. New York: Churchill Livingstone Inc.;1995. p. 1948-56.
3. Allos BM, Blaser MJ. *Campylobacter jejuni* and the expanding spectrum of related infections. Clin Infect Dis 1995;20:1092-9.
4. Dryden MS, Gabb RJ, Wright SK. Empirical treatment of severe acute community-acquired gastroenteritis with ciprofloxacin. Clin Infect Dis 1996;22:1019-25.
5. Skirrow MB, Blaser MJ, Blaser MJ, Smith PD, Ravdin JI, Greenberg HB, et al. editors. Infections of gastrointestinal tract. New York: Raven Press; 1995. p. 825-48.
6. Wistrom J, Norrby SR. Fluoroquinolones and bacterial enteritis, when and for whom? J Antimicrob Chemother 1995;36:23-39.
7. Piddock LJ. Quinolone resistance and *Campylobacter* spp. J Antimicrob Chemother 1995;36:891-8.
8. Adler-Mosca H, Lüthy-Hottenstein J, Martinetti Lucchini G, Burnens A, Altwegg M. Development of resistance to quinolones in five patients with campylobacteriosis treated with norfloxacin or ciprofloxacin. Eur J Clin Microbiol Infect Dis 1991;10:953-7.
9. Endtz HP, Ruijs GJ, van Klingeren B, Jansen WH, van der Reyden T, Mouton RP. Quinolone resistance in *Campylobacter* isolated from man and poultry following the introduction of fluoroquinolones in veterinary medicine. J Antimicrob Chemother 1991;27:199-208.
10. Smith KE, Besser JM, Hedberg CW, Leano FT, Bender JB, Wicklund JH, et al. Quinolone-resistant *Campylobacter jejuni* infections in Minnesota, 1992-1998. N Engl J Med 1999;340:1525-32.
11. Prescott JF, Baggot JD. Antimicrobial therapy in veterinary medicine. 2nd ed. Ames (IA): Iowa State University Press; 1993.
12. Taylor DE. In: Nachamkin I, Blaser MJ, Tompkins LS, editors. *Campylobacter jejuni* - current status and future trends. Washington: American Society for Microbiology; 1992. p. 74-86.
13. Yan W, Taylor DE. Characterization of erythromycin resistance in *Campylobacter jejuni* and *Campylobacter coli*. Antimicrob Agents Chemother 1991;35:1989-96.
14. Taylor DE, Ge Z, Purych D, Lo T, Hiratsuka K. Cloning and sequence analysis of two copies of a 23S rRNA gene from *Helicobacter pylori* and association of clarithromycin resistance with 23S rRNA mutations. Antimicrob Agents Chemother 1997;41:2621-8.
15. Trieber CA, Taylor DE. In: Mobley HLT, Nachamkin I, McGee D, editors. Abstracts and final program of the 10th International Workshop on *Campylobacter*, *Helicobacter* and related organisms. Baltimore: University of Maryland School of Medicine; 1999; Abstract CA6. p. 3.
16. Wang Y, Huang WM, Taylor DE. Cloning and nucleotide sequence of the *Campylobacter jejuni gyrA* gene and characterization of quinolone resistance mutations. Antimicrob Agents Chemother 1993;37:457-63.
17. Ruiz J, Goni P, Marco F, Gallardo F, Mirelis B, Jimenez De Anta T, et al. Increased resistance to quinolones in *Campylobacter jejuni*: a genetic analysis of *gyr*A gene mutations in quinolone-resistant clinical isolates. Microbiol Immunol 1998;42:223-6.
18. Gibreel A, Sjögren E, Kaijser B, Wretlind B, Sköld O. Rapid emergence of high-level resistance to quinolones in *Campylobacter jejuni* associated with mutational changes in *gyrA* and *parC*. Antimicrob Agents Chemother 1998;42:3276-8.
19. Gootz TD, Martin BA. Characterization of high-level quinolone resistance in *Campylobacter jejuni*. Antimicrob Agents Chemother 1991;35:840-5.
20. Charvalos E, Tselentis Y, Hamzehpour MM, Köhler T, Pechere J-C. Evidence for an efflux pump im multidrug-resistant *Campylobacter jejuni*. Antimicrob Agents Chemother 1995;39:2019-22.
21. van Diest J, de Jong A. Overview of quinolone usage for food-producing animals. In: Use of quinolones in food animals and potential impact on human health. Report and proceedings of a WHO meeting. Geneva: World Health Organization; 1999. p.97.
22. Antibiotic resistance in the European Union associated with therapeutic use of veterinary medicines. Report and qualitative risk assessment by the committee for veterinary medical products. London: The European Agency for the Evaluation of Medical Products; 1999.
23. Jacobs Reitsma WF, Kan CA, Bolder NM. The induction of quinolone resistance in *Campylobacter* in broilers by quinolone treatment. Lett Appl Microbiol 1994;19228-31.
24. Aarestrup FM, Nielsen EM, Madsen M, Engberg J. Antimicrobial susceptibility patterns of thermophilic *Campylobacter* spp. from humans, pigs, cattle, and broilers in Denmark. Antimicrob Agents Chemother 1997;41:2244-50.
25. Bager F, editor. Danmap 98 - Consumption of antimicrobial agents and occurrence of antimicrobial resistance in bacteria from food animals, food and humans in Denmark. Copenhagen, Denmark: Danish Zoonosis Centre; 1999. p.3.
26. Cabrita J, Rodriguez J, Braganca F, Morgado C, Pires I, Penha Goncalves A. Prevalence, biotypes, plasmid profile and antimicrobial resistance of *Campylobacter* isolates from wild and domestic animals from Northeast Portugal. J Appl Microbiol 1992;73:279-85.
27. Saenz Y, Zarazaga M, Lantero M, Gastanares MJ, Baquero F, Torres C. Antibiotic resistance in *Campylobacter* strains isolated from animals, foods, and humans in Spain in 1997-1998. Antimicrob Agents Chemother 2000;44:267-71.
28. Moore JE, Madden RH, Kerr JR, Wilson TS, Murphy PG. Erythromycin-resistant thermophilic *Campylobacter* species isolated from pigs [see comments]. Vet Rec 1996;138:306-7.
29. Nielsen EM, Nielsen NL. Serotypes and typability of *Campylobacter jejuni* and *Campylobacter coli* isolated from poultry products. Int J Food Microbiol 1999;46:199-205.
30. Endtz HP, Mouton RP, van der Reyden T, Ruijs GJ, Biever M, van Klingeren B. Fluoroquinolone resistance in *Campylobacter* spp. isolated from human stools and poultry products [letter] [see comments]. Lancet 1990;335:787.

31. Li CC, Chiu CH, Wu JL, Huang YC, Lin TY. Antimicrobial susceptibilities of *Campylobacter jejuni* and *coli* by using E-test in Taiwan. Scand J Infect Dis 1998;30:39-42.
32. Gaunt PN, Piddock LJ. Ciprofloxacin resistant *Campylobacter* spp. in humans: an epidemiological and laboratory study. J Antimicrob Chemother 1996;37:47-57.
33. Skirrow MB, Blaser MJ. In: Nachamkin I, Blaser MJ, Tompkins LS, editors. *Campylobacter jejuni*: current status and future trends. Washington: American Society for Microbiology; 1992. p. 3-8.
34. Neimann J, Engberg J, Mølbak K, Wegener HC. 4th World Congress on Foodborne Infections and Intoxications (Proceedings). Berlin: Federal Institute for Health Protection of Consumer and Veterinary Medicine; 1998. p. 298-303.
35. Engberg J, Gerner-Smidt P, Scheutz F, Nielsen EM, On SLW, Mølbak K. Water-borne *Campylobacter jejuni* infection in a Danish town—a 6-week continuous source outbreak. Clin Microbiol Infect 1998;4:648-56.
36. On SLW, Nielsen EM, Engberg J, Madsen M. Validity of *Sma*I-defined genotypes of *Campylobacter jejuni* examined by *Sal*I, *Kpn*I, and *Bam*HI polymorphisms: evidence of identical clones infecting humans, poultry, and cattle. Epidemiol Infect 1998;120:231-7.
37. Nielsen EM, Engberg J, Madsen M. Distribution of serotypes of *Campylobacter jejuni* and *C. coli* from Danish patients, poultry, cattle and swine. FEMS Immunol Med Microbiol 1997;19:47-56.
38. Doyle MP, Jones DM. In: Nachamkin I, Blaser MJ, Tompkins LS, editors. *Campylobacter jejuni* - current status and future trends. Washington: American Society for Microbiology; 1992. p. 45-8.
39. Piddock LJV. Working Paper 20.09. Geneva: World Health Organization; 1998. p. 1-9.
40. Gallardo F, Gascon J, Ruiz J, Corachan M, de Anta MTJ, Vila J. *Campylobacter jejuni* as a cause of traveler's diarrhea: clinical features and antimicrobial susceptibility. J Travel Med 1998;5:23-6.
41. Mattila L, Peltola H, Siitonen A, Kyronseppa H, Simula I, Kataja M. Short-term treatment of traveler's diarrhea with norfloxacin: a double-blind, placebo-controlled study during two seasons. Clin Infect Dis 1993;17:779-82.
42. Friedman CR, Yang S, Rocourt J, Stamey K, Vugia D, Marcus R, et al. In: 36th annual meeting of the Infectious Diseases Society of America - Program and Abstracts. Denver, Colorado: The Infectious Diseases Society of America; 1998; Abstract 545 Fr, p. 179.
43. Rautelin H, Renkonen OV, Kosunen TU. Emergence of fluoroquinolone resistance in *Campylobacter jejuni* and *Campylobacter coli* in subjects from Finland. Antimicrob Agents Chemother 1991;35:2065-9.
44. Sjögren E, Kaijser B, Werner M. Antimicrobial susceptibilities of *Campylobacter jejuni* and Campylo*bacter coli* isolated in Sweden: a 10-year follow-up report. Antimicrob Agents Chemother 1992;36:2847-9.
45. Sjögren E, Lindblom GB, Kaijser B. Norfloxacin resistance in *Campylobacter jejuni* and *Campylobacter coli* isolates from Swedish patients. J Antimicrob Chemother 1997;40:257-61.
46. Engberg J, Andersen S, Skov R, Aarestrup FM, Gerner-Smidt P. Comparison of two agar dilution methods and three agar diffusion methods including the E-test for antibiotic susceptibility testing of thermophilic *Campylobacter* species. Clin Microbiol Infect 1999;5:580-4.
47. Orr KE, Lightfoot NF, Sisson PR, Harkis BA, Tveddle JL, Boyd P, et al. Direct milk excretion of *Campylobacter jejuni* in a dairy cow causing cases of human enteritis. Epidemiol Infect 1995;114:15-24.
48. Pearson AD, Greenwood MH, Donaldson J, Healing TD, Jones DM, Shahamat M, et al. Continuous source outbreak of campylobacteriosis traced to chicken. J Food Prot 2000;63:309-14.
49. Owen RJ, Leeton S. Restriction fragment length polymorphism analysis of the flaA gene of *Campylobacter jejuni* for subtyping human, animal and poultry isolates. FEMS Microbiol Lett 1999;176:345-50.
50. Funke G, Baumann R, Penner JL, Altwegg M. Development of resistance to macrolide antibiotics in an AIDS patient treated with clarithromycin for *Campylobacter jejuni* diarrhea. Eur J Clin Microbiol Infect Dis 1994;13:612-5.
51. Segreti J, Gootz TD, Goodman LJ, Parkhurst GW, Quinn JP, Martin BA, et al. High-level quinolone resistance in clinical isolates of *Campylobacter jejuni*. J Infect Dis 1992;165:667-70.
52. Tee W, Mijch A. *Campylobacter jejuni* bacteremia in human immunodeficiency virus (HIV)-infected and non-HIV-infected patients: comparison of clinical features and review. Clin Infect Dis 1998;26:91-6.
53. Ellis-Pegler RB, Hyman LK, Ingram RJ, McCarthy M. A placebo controlled evaluation of lomefloxacin in the treatment of bacterial diarrhoea in the community. J Antimicrob Chemother 1995;36:259-63.
54. Molina J, Casin I, Hausfater P, Giretti E, Welker Y, Decazes J, et al. *Campylobacter* infections in HIV-infected patients: clinical and bacteriological features. AIDS 1995;9:881-5.
55. Chatzipanagiotou S, Papavasiliou E, Malamou Lada E. Isolation of *Campylobacter jejuni* strains resistant to nalidixic acid and fluoroquinolones from children with diarrhea in Athens, Greece [letter]. Eur J Clin Microbiol Infect Dis 1993;12:566-8.
56. Reina J, Ros MJ, Serra A. Susceptibilities to 10 antimicrobial agents of 1,220 *Campylobacter* strains isolated from 1987 to 1993 from feces of pediatric patients. Antimicrob Agents Chemother 1994;38:2917-20.
57. Sanchez R, Fernandez Baca V, Diaz MD, Munoz P, Rodriguez Creixems M, Bouza E. Evolution of susceptibilities of *Campylobacter* spp. to quinolones and macrolides. Antimicrob Agents Chemother 1994;38:1879-82.
58. Feierl G, Berghold C, Furpass T, Marth E. Further increase in ciprofloxacin-resistant *Campylobacter jejuni/coli* in Styria, Austria. Clin Microbiol Infect 1999;5:59-60.
59. Gaudreau C, Gilbert H. Antimicrobial resistance of clinical strains of *Campylobacter jejuni* subsp. *jejuni* isolated from 1985 to 1997 in Quebec, Canada. Antimicrob Agents Chemother 1998;42:2106-8.

60. Gaudreau C, Gilbert H. In: Mobley HLT, Nachamkin I, McGee D, editors. Abstracts and final program of the 10th International Workshop on *Campylobacter*, *Helicobacter* and Related Organisms. Baltimore: University of Maryland School of Medicine; 1999. Abstract CA3, p. 2.
61. Bager F, editor. DANMAP 97 - Consumption of antimicrobial agents and occurrence of antimicrobial resistance in bacteria from food animals, food and humans in Denmark. Copenhagen: Danish Zoonosis Centre; 1998. p.3.
62. Bager F, editor. DANMAP 99 - Consumption of antimicrobial agents and occurrence of antimicrobial resistance in bacteria from food animals, food and humans in Denmark. Copenhagen: Danish Zoonosis Centre; 2000.
63. Hänninen ML, Pajarre S, Klossner ML, Rautelin H. Typing of human *Campylobacter jejuni* isolates in Finland by pulsed-field gel electrophoresis. J Clin Microbiol 1998;36:1787-9.
64. Megraud F. Les infections à *Campylobacter* en France (1986-1997). Bulletin Epidémiologique Annuel 1998;2:83-4.
65. Varga J, Fodor L. Biochemical characteristics, serogroup distribution, antibiotic susceptibility and age-related significance of *Campylobacter* strains causing diarrhoea in humans in Hungary. Zentralbl Bakteriol 1998;288:67-73.
66. Itoh T, Tadano K, Obata H, Shingaki K, Kai A, Saito K, et al. Emergence of quinolone-resistance in clinical isolates of *Campylobacter jejuni* in Japan. In: Newell DG, Ketley J, Feldman RA, editors. 8th International Workshop on Campylobacters, Helicobacters and Related Organisms. Abstracts from the meeting held at Winchester, United Kingdom, 10th-13th July 1995. New Haw, Addlestone, England: Central Veterinary Laboratory; 1995. p. 83.
67. Piersimoni C, Crotti D, Nista D, Bornigia G, de Sio G. In: Newell DG, et al., editors. 8th International Workshop on Campylobacters, Helicobacters and Related Organisms. Abstracts from the meeting held at Winchester, United Kingdom, 10th - 13th July 1995. New Haw, Addlestone, England: Central Veterinary Laboratory; 1995; p. 88.
68. Crotti D, Medori MC, Fonzo G, Del Sante M, Silvestrini R. Clinical microbiology of Campylobacter enteritis in our experience. Clin Microbiol Infect 1999;7(Suppl. 3):267.
69. Crotti D, Fonzo G, D'Annibale ML, Medori, MC Luzzi I, Mobley HLT, et al., In: Mobley HLT, Nachamkin I, McGee D, editors. Abstracts and final program of the 10th International Workshop on *Campylobacter*, *Helicobacter* and Related Organisms. Baltimore: University of Maryland School of Medicine; 1999. Abstract CA7. p. 4.
70. Dowling J, MacCulloch D, Morris AJ. Antimicrobial susceptibility of *Campylobacter* and *Yersinia enterocolitica* isolates [letter]. N Z Med J 1998;111:281.
71. Lim YS, Tay L. A one-year study of enteric *Campylobacter* infections in Singapore. J Trop Med Hyg 1992;95:119-23.
72. Navarro F, Miro E, Mirelis B, Prats G. *Campylobacter* spp. antibiotic susceptibility [letter; comment]. J Antimicrob Chemother 1993;32:906-7.
73. Hoge CW, Gambel JM, Srijan A, Pitarangsi C, Echeverria P. Trends in antibiotic resistance among diarrheal pathogens isolated in Thailand over 15 years. Clin Infect Dis 1998;26:341-5.
74. Murphy GS, Jr, Echeverria P, Jackson LR, Arness MK, LeBron C, Pitarangsi C. Ciprofloxacin- and azithromycin-resistant *Campylobacter* causing traveler's diarrhea in U.S. troops deployed to Thailand in 1994. Clin Infect Dis 1996;22:868-9.
75. Frost JA, Thwaites RT. Drug resistance in *C. jejuni, C. coli* and *C. lari* isolated from humans in Wales and North West England during 1997. Working Paper 20.10b. Geneva: World Health Organization; 1998.
76. Baker CN. The E-Test and *Campylobacter jejuni*. Diagn Microbiol Infect Dis 1992;15:469-72.
77. National antimicrobial resistance monitoring system NARMS - 1997 annual report revised. Atlanta: Centers for Disease Control and Prevention; 1998.
78. Nachamkin I. Antimicrobial susceptibility of *Campylobacter jejuni* and *Campylobacter coli* to ciprofloxacin, erythromycin and tetracycline from 1982 to 1992. Med Microbiol Lett 1992;2:300-5.
79. Hirschl AM, Wolf D, Berger J, Rotter ML. In vitro susceptibility of *Campylobacter jejuni* and *Campylobacter coli* isolated in Austria to erythromycin and ciprofloxacin. Zentralbl Bakteriol 1990;272:443-7.
80. Feierl G, Pschaid A, Sixl B, Marth E. Increase of ciprofloxacin resistance in *Campylobacter* species in Styria, Austria. Int J Med Microbiol Virol Parasitol Infect Dis 1994;281:471-4.
81. Stobberingh E, van den Bogaard A, Mevius D, Endtz H. Examples of *in-vitro* quinolone resistance prevalence trends in humans and animal isolates of food-borne *Salmonella* and *Campylobacter*. Working Paper 20.09. Geneva: World Health Organization; 1998.
82. Perez Trallero E, Urbieta M, Lopategui CL, Zigorraga C, Ayestaran I. Antibiotics in veterinary medicine and public health [letter; comment]. Lancet 1993;342:1371-2.
83. Reina J. Resistance to fluoroquinolones in *Salmonella* non-typhi and *Campylobacter* spp. [letter; comment]. Lancet 1992;340:1035-6.
84. Kuschner RA, Trofa AF, Thomas RJ, Hoge CW, Pitarangsi C, Amato S, et al. Use of azithromycin for the treatment of *Campylobacter* enteritis in travelers to Thailand, an area where ciprofloxacin resistance is prevalent. Clin Infect Dis 1995;21:536-41.
85. Bowler I, Day D. Emerging quinolone resistance in campylobacters [letter; comment]. Lancet 1992;340:245.
86. McIntyre M, Lyons M. Resistance to ciprofloxacin in *Campylobacter* spp. [letter; comment]. Lancet 1993;341:188.
87. Bowler IC, Connor M, Lessing MP, Day D. Quinolone resistance and *Campylobacter* species [letter]. J Antimicrob Chemother 1996;38:315.
88. Sam WIC, Lyons MM, Waghorn DJ. Increasing rates of ciprofloxacin resistant *Campylobacter* [letter]. J Clin Pathol 1999;52:709.

89. Shah PM, Schafer V, Knothe H. Medical and veterinary use of antimicrobial agents: implications for public health. A clinician's view on antimicrobial resistance. Vet Microbiol 1993;35:269-74.
90. Reina J, Borrell N, Serra A. Emergence of resistance to erythromycin and fluoroquinolones in thermotolerant *Campylobacter* strains isolated from feces 1987-1991. Eur J Clin Microbiol Infect Dis 1992;11:1163-6.
91. Tee W, Mijch A, Wright E, Yung A. Emergence of multidrug resistance in *Campylobacter jejuni* isolates from three patients infected with human immunodeficiency virus. Clin Infect Dis 1995;21:634-8.
92. Threlfall EJ, Ward LR, Rowe B. Resistance to ciprofloxacin in non-typhoidal salmonellas from humans in England and Wales - the current situation. Clin Microbiol Infect 1999;5:130-4.
93. Mølbak K, Baggesen DL, Aarestrup FM, Ebbesen JM, Engberg J, Frydendahl K, et al. An outbreak of multidrug-resistant, quinolone-resistant *Salmonella* enterica serotype Typhimurium DT 104. N Engl J Med 1999;341:1420-5.
94. Pedersen KB, Aarestrup FM, Jensen NE, Bager F, Jensen LB, Jorsal SE, et al. The need for a veterinary antibiotic policy. Vet Rec 1999;(July 10):50-3.
95. Use of quinolones in food animals and potential impact on human health. Report of a WHO meeting, Geneva, Switzerland, 2-5 June 1998. Geneva: World Health Organization; 1998.
